# Aberrant over-expression of TRPM7 ion channels in pancreatic cancer: required for cancer cell invasion and implicated in tumor growth and metastasis

**DOI:** 10.1242/bio.20137088

**Published:** 2015-03-13

**Authors:** Nelson S. Yee, Abid A. Kazi, Qin Li, Zhaohai Yang, Arthur Berg, Rosemary K. Yee

**Affiliations:** 1 Division of Hematology-Oncology, Department of Medicine, Penn State College of Medicine, Program of Experimental Therapeutics, Penn State Hershey Cancer Institute, Penn State Milton S. Hershey Medical Center, Pennsylvania State University, Hershey, PA 17033, USA; 2 Division of Anatomic Pathology, Department of Pathology, Penn State College of Medicine, Penn State Milton S. Hershey Medical Center, Pennsylvania State University, Hershey, PA 17033, USA; 3 Division of Biostatistics and Bioinformatics, Department of Public Health, Penn State College of Medicine, Pennsylvania State University, Hershey, PA 17033, USA; 4 Schreyer Honors College, Pennsylvania State University, University Park, PA 16802, USA, Penn State Harrisburg School of Humanities, Pennsylvania State University, Middletown, PA 17057, USA

**Keywords:** TRPM7, Ion channel, Pancreatic cancer, Invasion, Biomarker, Target

## Abstract

Our previous studies in zebrafish development have led to identification of the novel roles of the transient receptor potential melastatin-subfamily member 7 (TRPM7) ion channels in human pancreatic cancer. However, the biological significance of TRPM7 channels in pancreatic neoplasms was mostly unexplored. In this study, we determined the expression levels of TRPM7 in pancreatic tissue microarrays and correlated these measurements in pancreatic adenocarcinoma with the clinicopathological features. We also investigated the role of TRPM7 channels in pancreatic cancer cell invasion using the Matrigel^TM^-coated transwell assay. In normal pancreas, TRPM7 is expressed at a discernable level in the ductal cells and centroacinar cells and at a relatively high level in the islet endocrine cells. In chronic pancreatitis, pre-malignant tissues, and malignant neoplasms, there is variable expression of TRPM7. In the majority of pancreatic adenocarcinoma specimens examined, TRPM7 is expressed at either moderate-level or high-level. Anti-TRPM7 immunoreactivity in pancreatic adenocarcinoma significantly correlates with the size and stages of tumors. In human pancreatic adenocarcinoma cells in which TRPM7 is highly expressed, short hairpin RNA-mediated suppression of *TRPM7* impairs cell invasion. The results demonstrate that TRPM7 channels are over-expressed in a proportion of the pre-malignant lesions and malignant tumors of the pancreas, and they are necessary for invasion by pancreatic cancer cells. We propose that TRPM7 channels play important roles in development and progression of pancreatic neoplasm, and they may be explored as clinical biomarkers and targets for its prevention and treatment.

## Introduction

Pancreatic adenocarcinoma is the most common malignant tumor of the pancreas, and it is almost uniformly fatal . The incidence of pancreatic adenocarcinoma has been rising, and its prognosis is generally dismal (Siegel et al., 2014). For most of the patients, pancreatic adenocarcinoma is typically diagnosed at locally advanced or metastatic stages, at which the tumor is marginally responsive to the conventional therapeutics. Novel strategies for developing effective and safe treatment demand identification and functional characterization of the genetic alterations involved in pancreatic carcinogenesis. Histopathologic and genetic analyses have elucidated the biological events during transformation of pancreatic epithelia into pancreatic intra-epithelial neoplasms (PanINs) and their progression to invasive adenocarcinoma. These events are associated with oncogene-induced senescence, followed by aberrant regulation of developmental pathways, tumor suppressor genes, and oncogenes, as well as epigenetic alterations (Chun and Yee, 2010; Hasanali et al., 2012; Yee, 2011; Yee, 2013; Yee et al., 2003; Zhou et al., 2011). Further studies that focus on the mechanisms underlying these pathogenetic events will help facilitate development of biomarkers and therapy targeting the molecular phenotype of individual pancreatic tumor. Increasing evidence has implicated fundamental roles of ion channels in human cancers. Their identities and functional roles in have recently been discovered in pancreatic cancer; in particular, the transient receptor potential (TRP) channels TRPM7 and TRPM8.

Ion channels are biological membrane proteins that control flow of cations and anions, resulting in modulation of cytoplasmic signaling and cellular responses (Yee, 2012). The TRP family ion channels share common architectural features and each subfamily is characterized by unique structural motifs (Nilius and Owsianik, 2011). Members of the TRP melastatin (TRPM) subfamily have been elucidated in various human malignancies, including pancreatic cancer (Yee, 2012; Yee et al., 2012a). We have discovered the regulatory roles of the zebrafish ortholog of mammalian TRPM7 in development of exocrine pancreas, and we subsequently identified the expression and functions TRPM7 and TRPM8 in human pancreatic cancer (Yee, 2013; Yee et al., 2012a; Yee et al., 2013; Yee and Yee, 2013; Yee et al., 2010; Yee et al., 2012b; Yee et al., 2011). TRPM7 channels are permeable to cations, and they possess intrinsic protein serine/threonine kinase activity. TRPM7 is ubiquitously expressed, and it is essential for a variety of biological and physiological processes as well as embryonic development (Yee et al., 2014). Expression of TRPM7 is increased in a panel of human pancreatic adenocarcinoma cells and tissues (Yee et al., 2012a; Yee et al., 2013; Yee and Yee, 2013; Yee et al., 2011). In culture studies using human pancreatic adenocarcinoma cell lines, TRPM7 channels have been shown to be necessary for maintaining proliferation and preventing replicative senescence (Yee et al., 2012a; Yee et al., 2012b; Yee et al., 2011). However, the expression and significance of TRPM7 in normal and various pre-malignant and malignant pancreatic tissues have not been examined in detail. Whether TRPM7 channels are required for pancreatic cancer cell invasion remains to be explored.

In this study, we aim to determine the significance of TRPM7 in pancreatic cancer by immunohistochemical analysis of its expression in pancreatic tissues and examine the role of TRPM7 in cancer cell invasion. The results indicate that TRPM7 is aberrantly over-expressed in pre-malignant tissues and in various types of pancreatic neoplasms. There is a statistically significant correlation between the expression levels of TRPM7 in pancreatic adenocarcinoma and the size and stages of tumors. Moreover, TRPM7 is required for pancreatic cancer invasion. These data have led us to hypothesize that TRPM7 channels play functional roles in the development and progression of pancreatic cancer. We propose that TRPM7 can be further explored as clinical biomarkers and targets for prevention and treatment of this highly lethal disease.

## Results

### Expression of TRPM7 in normal and pre-malignant pancreatic tissues

Microarrays containing 366 specimens of human pancreatic tissues were evaluated for expression of TRPM7 by immunohistochemistry (IHC) using specific antibodies raised against TRPM7. The specimens include normal tissues of pancreas, chronic pancreatitis, pre-malignant neoplasms, and various types of malignant tumors. The proportions of the different types of histopathology of pancreatic tumors under examination are representative of what is typically seen in clinical practice. As shown in Table 1, most of the pancreatic tumors present in the tissue microarrays are adenocarcinoma, which is the most common type of pancreatic cancer . Besides, there are relatively few metastatic tumors as compared to primary pancreatic adenocarcinoma (Table 2). This can be attributed to the fact that primary pancreatic tumors available for IHC were mostly obtained by surgical resection, whereas tumors in the metastatic sites are typically biopsied using fine needle aspiration. However, the pancreatic tissue from each patient was processed as triplicates, and the IHC analysis of TRPM7 is consistent among the triplicate samples of each specimen, thus supporting the validity of the data.

**Table 1.**
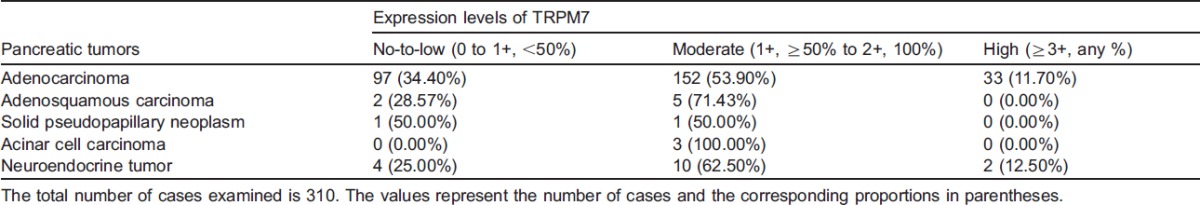
Expression of TRPM7 in malignant pancreatic tumors as indicated by the intensity and the percentage of positive cells

**Table 2.**
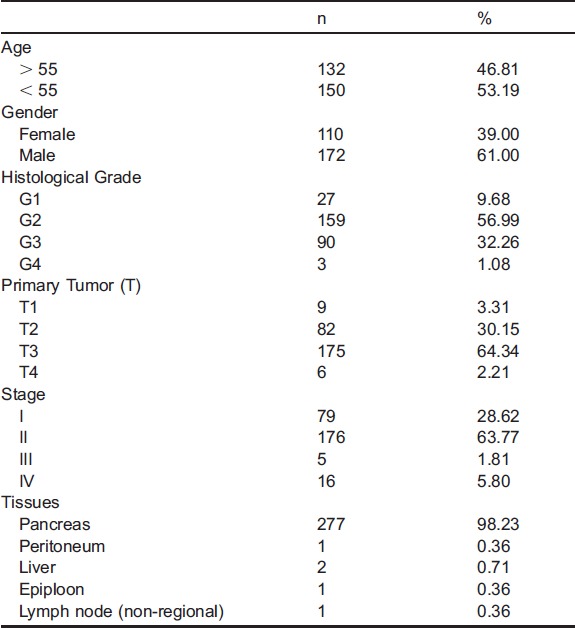
Clinicopathological features of pancreatic adenocarcinoma in the tissue microarrays

**Fig. 1.**
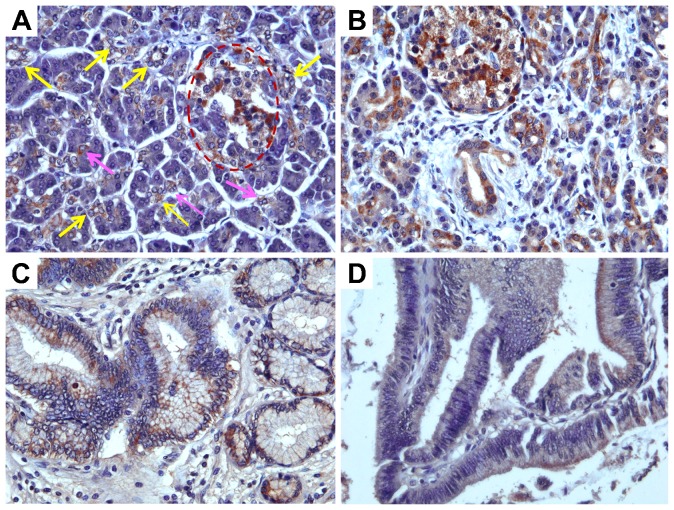
Immunohistochemical analysis of TRPM7 in non-malignant pancreatic tissues. (A) Normal pancreatic tissues. (B) Chronic pancreatitis. (C) Pancreatic intra-epithelial neoplasm. (D) Intraductal papillary mucinous neoplasm. Original magnification ×400. In panel A, yellow arrows point at ducts, and pink arrows point at centroacinar cells; the islet is surrounded with red dotted lines.

In the normal tissues of exocrine pancreas, anti-TRPM7 immunoreactivity was observed in the ductal and centroacinar cells, but minimal, if any, in the acinar cells (Fig. 1A). In the ductal epithelia and centroacinar cells, expression of TRPM7 is relatively low and mostly cytoplasmic with accentuation on the luminal plasma membrane. In the endocrine cells of the pancreatic islets, there is a moderate level of anti-TRPM7 immunoreactivity with uneven distribution in the cytoplasm (Fig. 1A). In chronic pancreatitis, the expression level of TRPM7 is relatively high; anti-TRPM7 immunoreactivity in the cytoplasm of the pancreatic ductal and acinar cells is readily detectable (Fig. 1B). In pancreatic intra-epithelial neoplasms (PanINs) and intraductal papillary mucinous neoplasm (IPMN), there is a moderate level of TRPM7 expression that appears to be localized on the plasma membrane (Fig. 1C,D). Thus, TRPM7 is differentially expressed in different types of epithelia in normal pancreatic tissues, and its expression appears elevated in chronically inflamed and pre-malignant tissues with localization on the plasma membrane.

### Expression of TRPM7 in malignant tumors of pancreas

**Fig. 2.**
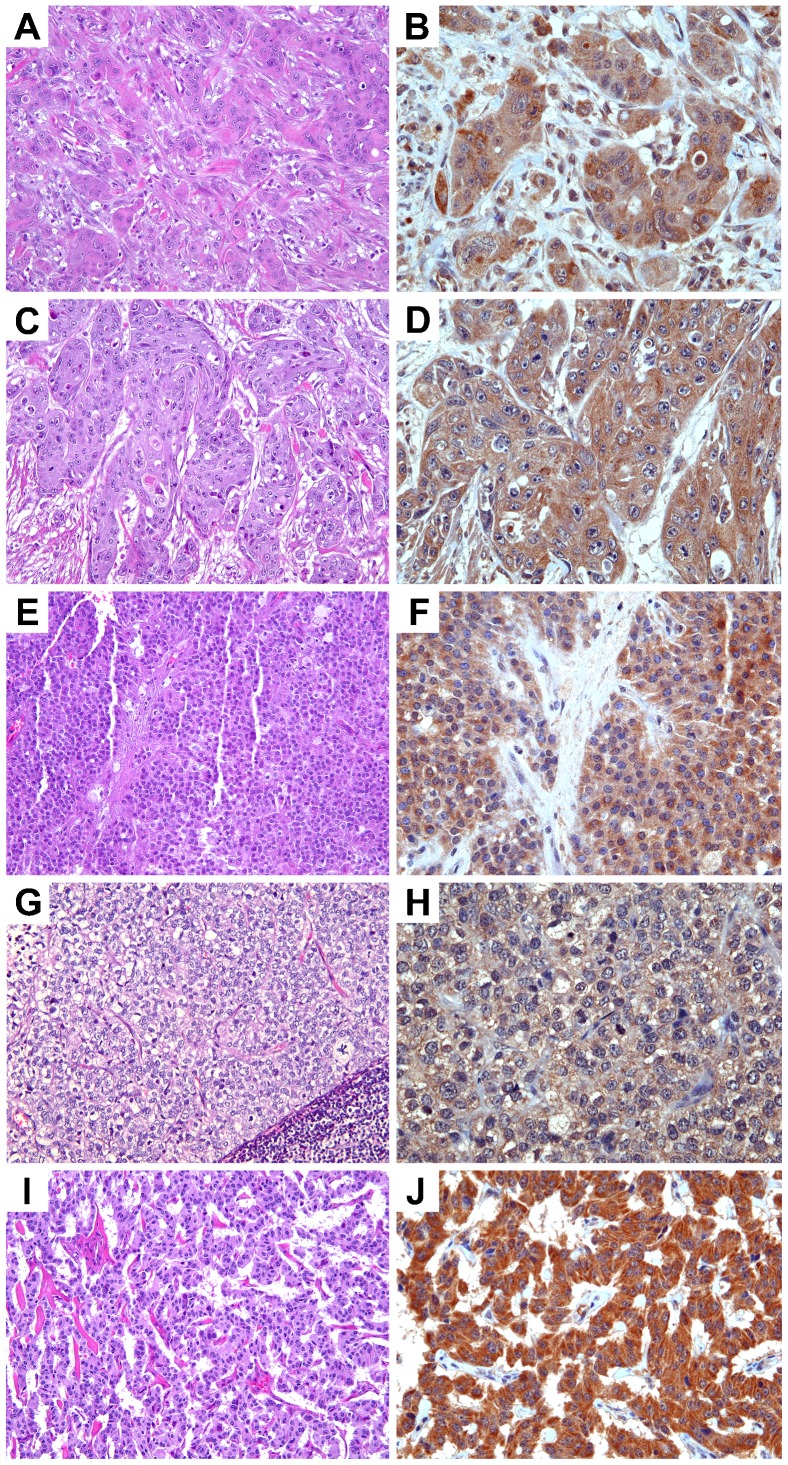
Immunohistochemical analysis of TRPM7 in malignant pancreatic tumors. (A,B) Adenocarcinoma. (C,D) Adenosquamous carcinoma. (E,F) Solid pseudopapillary neoplasm. (G,H) Acinar cell carcinoma. (I,J) Neuroendocrine tumor. H&E, original magnification ×200 (A,C,E,G,I). Immunohistochemistry using anti-TRPM7 antibodies, original magnification ×400 (B,D,F,H,J).

Anti-TRPM7 immunoreactivity was examined in pancreatic adenocarcinoma, adenosquamous carcinoma, solid pseudopapillary neoplasm, acinar cell carcinoma, and neuroendocrine tumor. TRPM7 is expressed at a moderate to high level in the majority of pancreatic adenocarcinoma cases (Fig. 2A,B). Notably, anti-TRPM7 immunoreactivity in pancreatic adenocarcinoma is accentuated towards the plasma membrane (Fig. 2B). In pancreatic adenosquamous carcinoma and solid pseudopapillary neoplasm, TRPM7 is expressed at low to moderate levels (Fig. 2C–F). In contrast to normal pancreatic acinar cells, which have no appreciable expression of TRPM7 (Fig. 1A), there is moderate anti-TRPM7 immunoreactivity in all cases of acinar cell carcinoma examined (Fig. 2G,H). In pancreatic neuroendocrine tumors, moderate to high expression of TRPM7 was identified (Fig. 2I,J). For each histological type of pancreatic tumor, the proportions of samples with corresponding TRPM7 expression levels are described in Table 1. These data suggest that TRPM7 is aberrantly over-expressed in various types of malignant pancreatic neoplasms.

### Expression of TRPM7 in pancreatic adenocarcinoma correlates with the size and stages of tumors

**Fig. 3.**
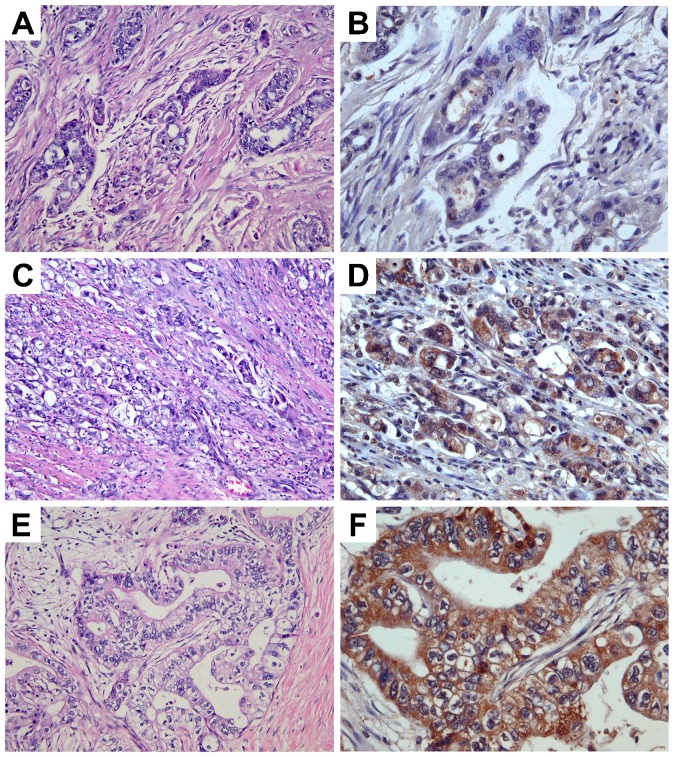
Expression levels of TRPM7 in pancreatic adenocarcinoma. Images in A,C,E (original magnification ×200) represent the H&E sections of the same tumor as the TRPM7 immunohisto-chemical staining in B,D,F (original magnification ×400), respectively. Expression levels of TRPM7: (B) none-to-low; (D) moderate; (F) high.

**Fig. 4.**
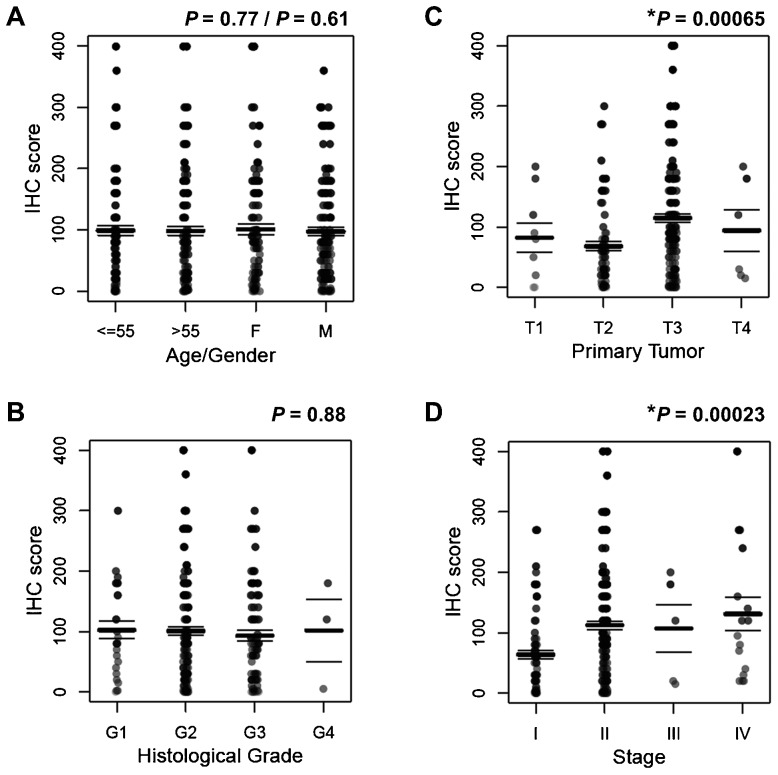
Anti-TRPM7 immunoreactivity in pancreatic adenocarcinoma positively correlates with tumor size and stages. The distribution of the IHC score for expression of TRPM7 is displayed against (A) age or gender, (B) histological grade, (C) primary tumor [size], and (D) stage. The total number of specimens of pancreatic adenocarcinoma examined is 282. (C) Polyserial correlation between the primary tumor size (T) and the log-transformed IHC score is 0.24. (D) Polyserial correlation between the tumor stages and the log-transformed IHC score is 0.26. *P-values indicate statistically significant positive association of primary tumor size (T) or tumor stages with the anti-TRPM7 IHC score. The bars represent mean and s.e.m.

To determine the clinical significance of TRPM7 in pancreatic adenocarcinoma, we further analyzed the expression of TRPM7 in detail and correlated it with the clinical and pathological features. The characteristics of the 282 cases of pancreatic adenocarcinoma present in the tissue microarrays are listed in Table 2. On the basis of the percent coverage by IHC separated by staining intensity and tumor stages, the expression levels of TRPM7 in pancreatic adenocarcinoma are displayed (Table 3). The majority of the pancreatic adenocarcinoma specimens (66%) exhibit moderate to high anti-TRPM7 immunoreactivity (Fig. 3). A positive correlation between the expression levels of TRPM7 and the primary tumor size and tumor stages was identified (Fig. 4). In particular, there is significantly higher anti-TRPM7 immunoreactivity in the primary tumor at T3 than at T2 (*P* = 0.00044). Similarly, the expression levels of TRPM7 in stage II tumors or stage IV tumors are significantly higher than in stage I (*P* = 0.00017 and *P* = 0.03, respectively). There is no significant correlation between the expression levels of TRPM7 and the age and gender of the patients or histological grade (Fig. 4).

**Table 3.**
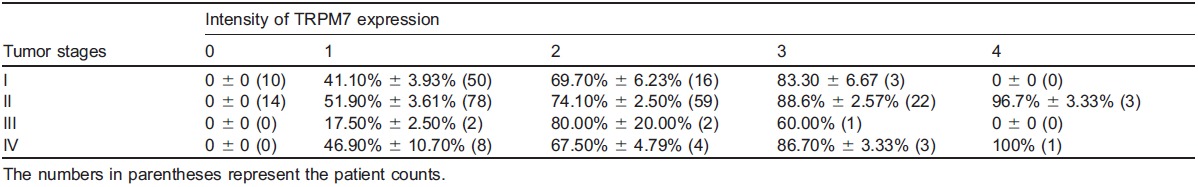
The percent coverage ± standard error for anti-TRPM7 immunoreactivity in pancreatic adenocarcinoma with respect to expression intensity and tumor stages

Among the 282 cases of pancreatic adenocarcinoma described above, 16 cases are pancreatic tumors that metastasized to other organs (Table 2). Elevated expression of TRPM7 in metastatic tumors (n = 16) as compared to that in non-metastatic pancreatic adenocarcinoma (stages I, II, and III combined, n = 266) was present but statistically marginal (*P* = 0.1). Sub-group analysis indicates that anti-TRPM7 immunoreactivity in the primary pancreatic tumor that metastasized (n = 11) tends to be higher than that in non-metastatic pancreatic tumors (n = 266) (*P* = 0.06). Among the metastatic tumors, there is no significant difference between anti-TRPM7 immunoreactivity in the primary tumors and that in the metastasized tumors. Taken together, these results strongly suggest that aberrantly over-expressed TRPM7 is associated with pancreatic tumor growth and possibly metastasis.

### TRPM7 is necessary for pancreatic cancer cell invasion

**Fig. 5.**
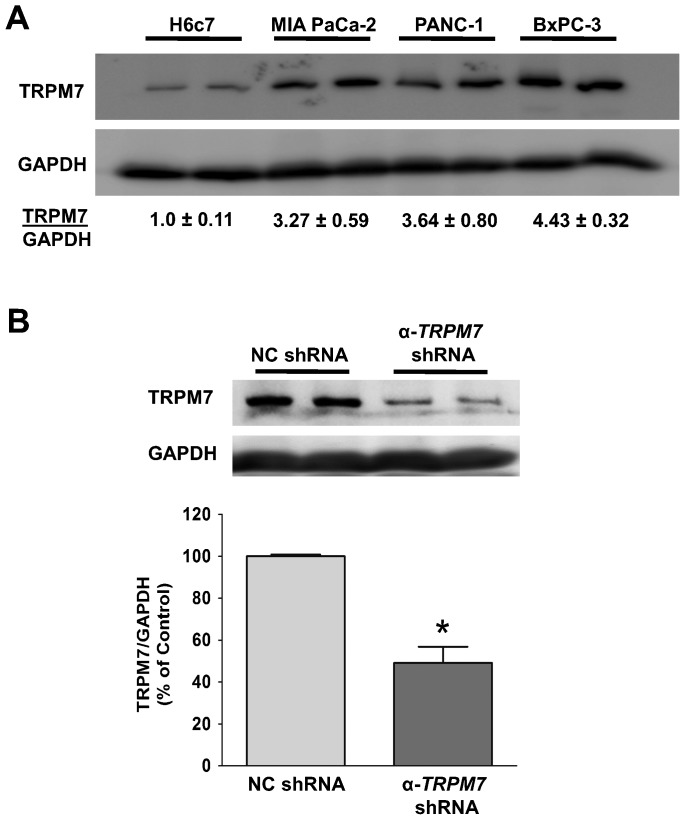
TRPM7 is over-expressed in pancreatic adenocarcinoma cells, and its expression can be down-regulated by shRNA. (A) Cell lysates of each cell line were analyzed for the protein levels of TRPM7 by immunoblotting. The relative amount of TRPM7 and GAPDH protein in each cancer cell line was compared to that in H6c7 as indicated (mean ± standard error). (B) Immunoblotting analysis of TRPM7 protein in BxPC-3 cells transfected with either non-targeting control (NC) shRNA or anti-*TRPM7* shRNA. The relative amount of TRPM7 and GAPDH protein in each group of cells is expressed as % of control (NC shRNA). Each column represents the mean ± standard error of three experiments with duplicate samples in each experiment. * indicates statistically significant difference with *P*<0.05.

To further understand the significance of the aberrantly over-expressed TRPM7 in pancreatic cancer, we investigated the role of TRPM7 in cell invasion by reducing its expression. In a previous report, we provided evidence that the levels of *TRPM7* mRNA are relatively high in human pancreatic adenocarcinoma cell lines including BxPC-3, PANC-1, MIA PaCa-2, PL45, and Capan-1 (Yee et al., 2011). Moreover, we demonstrated that BxPC-3 and PANC-1 cells with small interfering RNA (siRNA)-mediated repression of *TRPM7* exhibited significant reduction of proliferative capability and induction of replicative senescence (Yee et al., 2012b; Yee et al., 2011). Consistent with our previous studies (Yee et al., 2011), the relative level of *TRPM7* protein in BxPC-3, PANC-1, and MIA PaCa-2 are relatively high as compared to that in the non-cancerous pancreatic ductal epithelia H6c7 (Fig. 5A). In BxPC-3 cells, anti-*TRPM7* short hairpin (shRNA) down-regulated the protein levels of *TRPM7* by about 50%, as compared to those transfected with the control non-targeting shRNA (Fig. 5B).

To assay for cell invasion, BxPC-3 cells transfected with anti-*TRPM7* shRNA or control shRNA were seeded in transwell inserts with filters coated with a solubilized tumor-associated basement membrane matrix (Matrigel^TM^). Fetal bovine serum (FBS) was used as a chemoattractant for cell invasion. Prior to seeding the cells, the viability of the cells transfected with either control shRNA or anti-*TRPM7* shRNA was almost 100% as determined by trypan blue exclusion.

**Fig. 6.**
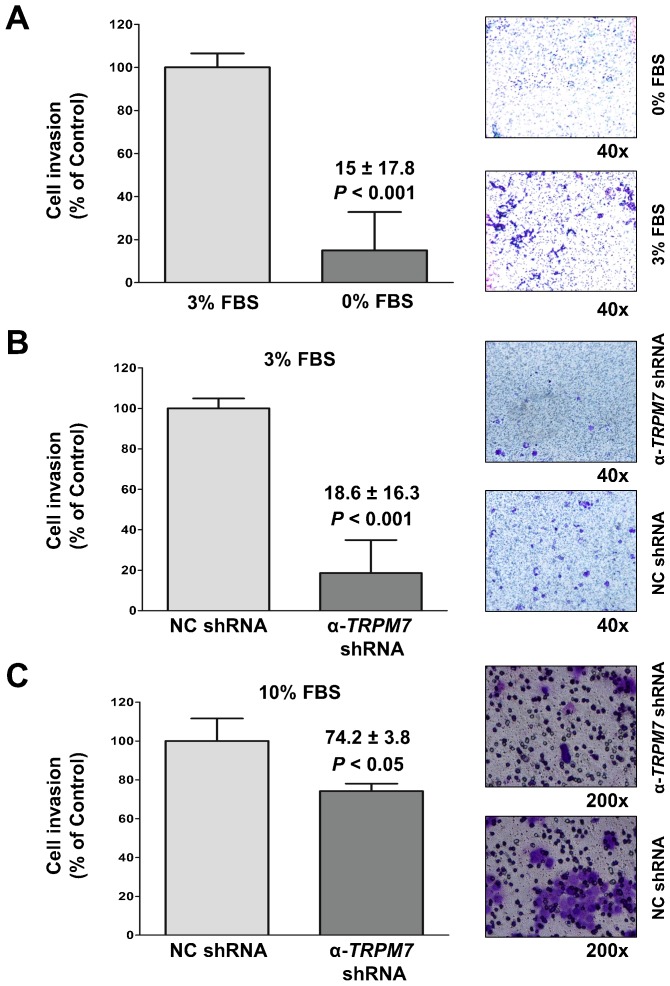
ShRNA-mediated silencing of *TRPM7* impaired invasion in pancreatic adenocarcinoma cells. (A) Non-transfected BxPC-3 cells assayed for invasion using the trans-well assay in the presence of 3% FBS or no FBS. (B,C) BxPC-3 cells transfected with non-targeting control (NC) shRNA or anti-*TRPM7* shRNA analyzed for cell invasion in the presence of either 3% FBS (B) or 10% FBS (C). A representative image of the invaded cells stained with crystal violet is shown for each experimental group. Cell invasion is expressed as % control, and each column represents the mean ± standard error.

In the non-transfected BxPC-3 cells, cell invasion occurred in response to 3% FBS, while most of the cells failed to invade in the absence of serum (Fig. 6A). As compared to the control cells transfected with non-targeting shRNA, BxPC-3 cells with partially repressed *TRPM7* had impaired ability to migrate and invade in response to either 3% or 10% FBS (Fig. 6B,C). Similarly, another pancreatic adenocarcinoma cell line (MIA PaCa-2) expressing anti-*TRPM7* shRNA exhibited reduced ability of cell invasion (N.S.Y., personal observation). These results indicate that TRPM7 is required for invasion of the pancreatic cancer cells, and support a potential role of TRPM7 channels in tumor metastasis.

## Discussion

We have translated the embryological and genetic studies of exocrine pancreas organogenesis in zebrafish into identification of the TRPM7 ion channels in human pancreatic adenocarcinoma (Yee et al., 2012a; Yee and Yee, 2013; Yee et al., 2012b; Yee et al., 2011). However, the clinicopathological significance of TRPM7 in pancreatic neoplasms and the functional roles of TRPM7 in pancreatic cancer remain to be explored. In this study, we provide evidence that TRPM7 is variably expressed in different types of pancreatic tumors and aberrantly over-expressed in most of the 282 cases of pancreatic adenocarcinoma examined. The variable levels of TRPM7 expression in pancreatic adenocarcinoma may be related to the underlying tumor heterogeneity. Importantly, the expression levels of TRPM7 in pancreatic adenocarcinoma positively correlate with the tumor size and stage. Moreover, anti-TRPM7 immunoreactivity tends to be stronger in pancreatic adenocarcinoma that metastasizes than non-metastatic tumors. Consistent with the immunohistochemical data, in the pancreatic adenocarcinoma cell line with elevated expression of TRPM7, targeted silencing of *TRPM7* impairs the cancer cells ability of invasion. In agreement with these data, a recent study has shown relatively intense immunoreactivity against TRPM7 in 18 specimens of pancreatic adenocarcinoma, and that TRPM7 is required for migration of cancer cells (Rybarczyk et al., 2012). Taken together, the current data and our previous findings suggest an important role of TRPM7 ion channels in mediating the growth and metastasis of pancreatic cancer.

The relatively high anti-TRPM7 immunoreactivity in chronic pancreatitis, PanINs, IPMN, and pancreatic adenocarcinoma implies a contributory role of TRPM7 channels in pancreatic carcinogenesis. Chronic pancreatitis is considered a risk factor for development of pancreatic cancer (Dítě et al., 2012; Guerra et al., 2011; Guerra et al., 2007; Lowenfels et al., 1993); PanINs and IPMN are precursor lesions of pancreatic adenocarcinoma (Brugge et al., 2004; Cooper et al., 2013). In those pre-malignant and malignant pancreatic tissues, the elevated immunoreactivity against TRPM7 is associated with localization of the TRPM7 channels on the plasma membrane. It has been shown in vascular endothelia, functional TRPM7 channels accumulate on the plasma membrane with increase in TRPM7-mediated current in response to fluid flow-induced shear stress (Oancea et al., 2006). We speculate that the up-regulated and activated TRPM7 channels contribute to the development and progression of the pre-malignant lesions and invasive adenocarcinoma. In an attempt to understand the mechanism underlying the aberrant over-expression of TRPM7 in pancreatic adenocarcinoma, we analyzed genomic *TRPM7* by quantitative real-time PCR using primers directed against the sequence within intron 16 of *TRPM7*. We found that *TRPM7* DNA in the genome of three pancreatic adenocarcinoma cell lines previously shown to express elevated levels of *TRPM7* mRNA is not amplified relative to non-cancerous pancreatic ductal epithelia (Yee, N.S., data not shown). These suggest that mechanisms other than gene amplification may account for the aberrantly elevated expression of TRPM7 in pancreatic adenocarcinoma.

The positive correlation between TRPM7 expression in pancreatic adenocarcinoma and the tumor size and metastasis is consistent with the functional roles of TRPM7 in pancreatic cancer cells. We have previously reported that TRPM7 is essential for maintaining Mg^2+^-dependent proliferation and preventing replicative senescence in pancreatic adenocarcinoma cells (Yee et al., 2012b; Yee et al., 2011). In this study, we present evidence that shRNA-mediated silencing of *TRPM7* impairs the ability of pancreatic cancer cells to invade through tumor-associated extracellular matrix in response to 3% or 10% FBS. Consistent with these data, siRNA-induced down-regulation of *TRPM7* reduced pancreatic cancer cell migration in the presence of 20% FBS (Rybarczyk et al., 2012). Based on these observations, we hypothesize that the TRPM7 channels regulate cellular fluxes of Mg^2+^ and modulate mitogen-induced signaling, which mediate its control of growth and invasion in pancreatic cancer.

The mechanisms that mediate the various cellular effects of TRPM7 in pancreatic cancer remain to be determined. We speculate that TRPM7 and its regulated Mg^2+^ homeostasis modulate epidermal growth factor (EGF)-induced signaling pathways, resulting in cellular proliferation, survival, and migration/invasion of pancreatic cancer cells (Yee et al., 2012a). Studies in other cell systems have begun to shed light on the signaling mechanisms that mediate the functional roles of TRPM7. During zebrafish development, Mg^2+^-sensitive signaling involving Socs3a plays a crucial role in Trpm7-regulated pancreatic epithelial proliferation and exocrine pancreatic growth (Yee et al., 2011). In murine lymphocytes, STAT3-mediated signaling and the PI3K-mTOR pathway have been implicated in the proliferative effect of TRPM7 (Jin et al., 2008; Sahni and Scharenberg, 2008). In human lung cancer cells, ligand-activation of EGF receptor has been shown to induce cell migration with up-regulation of TRPM7 channels (Gao et al., 2011). It has been suggested that TRPM7, which has a conserved protein domain as in myosin II, is involved in the regulation of myosin II-based tension. Such TRPM7-controlled cellular mechanics in-turn regulates intercellular focal adhesion as well as mitogen-activated cell growth and proliferation (Middelbeek et al., 2012). These cellular responses may involve Mg^2+^-mediated activation of the alpha kinase of TRPM7 or direct phosphorylation of myosin II by TRPM7 (Vicente-Manzanares et al., 2009). Further studies are indicated to help establish the functional roles of TRPM7 channels and the associated mechanisms in the growth and progression of pancreatic neoplasm.

In conclusion, expression of TRPM7 is cell-type specific in adult human pancreatic tissues, and the TRPM7 ion channels are aberrantly expressed in various types of pancreatic neoplasms. Over-expression of TRPM7 in pancreatic adenocarcinoma positively correlates with increased tumor size and advanced tumor stages. These data suggest a contributory role of TRPM7 in tumor growth and invasion during pancreatic carcinogenesis, and this hypothesis is supported by the *in vitro* data demonstrating the requirement of TRPM7 for proliferation, migration, and invasion of pancreatic cancer cells. Continued research to understand the biology of TRPM7 channels will have important implications for exploitation of TRPM7 as biomarkers and targets with the hope of producing a positive impact towards personalized therapy and prevention of pancreatic cancer.

## Materials and Methods

### Pancreatic tissue microarrays

Tissue microarrays of human pancreas were obtained from US Biomax, Inc (Rockville, Maryland, USA). These tissues contain a variety of pancreatic tumors as well as benign and normal pancreatic tissues. Each specimen from an individual patient is present in triplicate on the slides being examined. Demographic data including age and gender, along with clinicopathological features, such as histopathology, histological grade, size of primary tumor, invasion, involvement of regional lymph node, and metastatic sites, are used for correlation with the expression levels of TRPM7. Tumor staging is based on the 7th edition of the American Joint Cancer Commission. The types of treatment received, therapeutic response, and data on survival that correspond to the pancreatic tissue microarrays are not available from US Biomax, Inc.

### Immunohistochemical analysis of TRPM7 expression

Hematoxylin and eosin (H&E) staining of the tissue microarray slides were performed using standard procedures. IHC analysis of TRPM7 expression was conducted using goat anti-human TRPM7 polyclonal IgG antibodies (Abcam®, Cambridge, Massachusetts, USA) at a 1:50 dilution. Following incubation with the primary antibodies, the tissues were incubated with horseradish peroxidase-conjugated anti-goat IgG (EnVision™+ System, DAKO, Carpinteria, California, USA). Color reaction using 3,3′-diaminobenzidine (DAKO) and counterstaining with hematoxylin (Richard-Allan Scientific®/VWR, Radnor, Pennsylvania, USA) were done to detect IHC signals. Tissue sections incubated with goat non-specific primary IgG antibodies or non-specific secondary anti-goat IgG antibodies were processed under the same conditions, and no TRPM7-specific immunoreactivity was detected (supplementary material Fig. S1).

Each histological section in the tissue microarrays is present as triplicates. All triplicates of each specimen were examined under a light microscope (BX40, Olympus, Tokyo, Japan) to determine the histopathological features and anti-TRPM7 immunoreactivity. The pathologist (Z.Y.) and the oncologist (N.S.Y.) were both blinded from the clinical and pathological data at the time of scoring IHC. Using a conventional semi-quantitative scoring system of 0 to 4+ for intensity and the estimated percentage of cells that express the protein, the expression level of TRPM7 was determined as follows: [0 to 1+ and <50%] no-to-low expression, [1+ and ≥ 50% to 2+ and 100%] moderate expression, and [≥ 3+ and any %] high expression. Digital images were acquired using a digital camera (DP12, Olympus) attached to the microscope and processed with Adobe® Photoshop® 7.

### Statistical analysis

The expression levels of TRPM7 as determined by IHC were scored by multiplying the intensity (range 0–4) and the proportion of positive cells (range 0–100%). The distribution of the IHC score against patients' age and gender, primary tumor size, histological grade, and tumor stage were shown along with the respective means, standard errors, and *P*-values. The statistical significance was determined using linear regression with the IHC score as the response variable. The ordered categorical variables (primary tumor size, histological grade, and tumor stage) were examined with an interval scale (1 to 4 for each variable). Hence, the *P*-values for the ordered categorical variables are analogous to a trend test, and the *P*-values for the binary variables (age > 55, gender) are analogous to a two-sample *t*-test. R (R Core Team, 2014) was employed for statistical analysis. The graphics for Fig. 4 was produced with the beeswarm package in R. Using the pairwise-t-test function in R with Bonferroni adjustments to the *P*-values to account for multiple comparisons, six pairwise comparisons were performed on the log-transformed IHC score data. Polyserial correlation between the ordinal variables of primary tumor size/stage and the log-transformed IHC score data was determined by using the polyserial function in the polycor library in R. Statistical significance was determined with a two-sided permutation test using 10^5^ permutations.

### Cell culture

The human pancreatic adenocarcinoma cell lines BxPC-3, PANC-1, and MIA PaCa-2 were purchased from the American Type Culture Collection (ATCC, Manassas, Virginia, USA) and cultured according to ATCC's instructions. The immortalized human pancreatic ductal epithelial cell line (H6c7) was kindly provided by Dr. Ming-Sound Tsao (University of Toronto) and cultured as described in the published literature (Furukawa et al., 1996). The cells were incubated at 37°C with humidified air containing CO_2_ at 5%. The cell lines were used no more than twenty passages, and the cells were periodically recovered from the frozen stocks stored in liquid nitrogen.

### Immunoblotting analysis

For analysis of TRPM7 protein levels, 2.5×10^6^ cells of each cell line were plated in a 10-cm cell culture dish (Corning, Corning, NY, USA) containing 15 ml medium with antibiotics and incubated for 48 h. Cell lysates were collected in RIPA buffer (Sigma-Aldrich®) fortified with a EDTA-free cocktail containing protease inhibitors (Roche Diagnostics, Indianapolis, Indiana, USA). The lysates were sonicated for 10 min and then kept on a rocker for 30 min at 4°C, followed by centrifugation at 14,000 ***g*** at 4°C for 10 min.

For each protein sample, equal volume was loaded in each lane and separated using 10% SDS-PAGE running overnight. The proteins were transferred to polyvinylidene fluoride membrane (Biotrace; PALL, Pensacola, Florida, USA), blocked in 5% non-fat dry milk, and then incubated overnight at 4°C with anti-human TRPM7 (rabbit polyclonal, 1:750 dilution) and anti-human GAPDH (mouse monoclonal, 1:2,500 dilution) antibodies (OriGene Technologies, Rockville, Maryland, USA), respectively. The membranes were rinsed with Tris-buffered saline containing 0.1% Tween 20, and then incubated with horse-radish peroxidase–conjugated goat anti-rabbit or goat anti-mouse secondary antibodies (Bethyl Laboratories, Montgomery, Texas, USA) at room temperature for 1 h. To remove excess secondary antibodies, the membranes were washed with Tris-buffered saline containing 0.1% Tween 20. Enhanced chemiluminescence (ECL plus, Amersham, Piscataway, New Jersey, USA) was used for detection of TRPM7 and GAPDH proteins according to the manufacturer's instructions. The chemiluminescent signals were processed by using the ProteinSimple Fluorchem M imaging system (Santa Clara, California, USA). GAPDH protein was assayed as internal control for loading equal amount of protein samples. The protein levels of TRPM7 were normalized with those of GAPDH by quantifying the protein levels in the original images using the ImageJ 1.6 software from the National Institutes of Health.

### RNA interference-mediated repression of *TRPM7*

Four different plasmids each containing a unique shRNA directed against human *TRPM7* and a plasmid containing non-targeting control shRNA were purchased from Superarray Biosciences (Qiagen, Valencia, California, USA) and tested for silencing of the *TRPM7* gene. Each plasmid carries a shRNA under U1 promoter control and green fluorescent protein (GFP) as the reporter gene. The sequences of the anti-*TRPM7* shRNA were: 5′-ATACCGGATTGGTTACAAGAT-3′; 5′-AGAGAGAATTCGTGTCACTTT-3′; 5′-ACAAGGTCATACTAAGCATTT-3′; 5′-CCAGATCTGAAGAGGAATGAT-3′. Non-targeting control shRNA: 5′-GGAATCTCATTCGATGCATAC-3′.

BxPC-3 cells at about 90% confluency in a 10-cm dish containing 10 ml OptiMem® medium were transfected with either 24 µg anti-*TRPM7* shRNA or non-targeting shRNA using 60 µl Lipofectamine™ 2000 (Invitrogen™, Life Technologies, Grand Island, New York, USA) and then incubated at 37°C for 5 h. The transfected cells were incubated in RPMI 1640 medium containing 10% FBS (Life Technologies) at 37°C for 24 h, sorted, and collected by flow cytometry for GFP with emission light at a 488-nm wavelength using BD FACSCalibur® (BD Biosciences, San Jose, California, USA). Using total RNA extracted from the GFP-sorted cells, *TRPM7* mRNA was analyzed by reverse transcription, followed by semi-quantitative polymerase chain reaction (PCR) as described (Yee et al., 2011). The TRPM7 protein levels in BxPC-3 cells transfected with anti-*TRPM7* shRNA or non-targeting control shRNA were analyzed as described above. Among the four anti-*TRPM7* shRNAs, the shRNA targeting the sequence (5′-AGAGAGAATTCGTGTCACTTT-3′) produced repression of *TRPM7* mRNA and protein to the greatest extent, and this shRNA was used for further experiment.

### Cell invasion assay

BxPC-3 cells transfected with anti-*TRPM7* shRNA or non-targeting control shRNA, and non-transfected cells, were cultured in RPMI 1640 medium containing 10% heat-inactivated FBS at 37°C for 24 h. The cells were washed in phosphate-buffered saline (PBS) and incubated in serum-free medium containing 0.1% bovine serum albumin (Sigma®) for another 48 h. The cells were then seeded at 3×10^4^ cells in 1 ml medium in each well of a 6-well cell culture dish (Corning). Trans-well inserts with 8 µm filters (Greiner Bio-One, VWR, Radnor, Pennsylvania, USA) coated with 10% Matrigel^TM^ (BD Biosciences) were used as a barrier for invasion. As a chemoattractant, 2.5 ml of 3% FBS or 10% FBS was placed in the lower chamber. The cells were incubated at 37°C for 24 h and analyzed for cell invasion.

The cells on the lower side of the insert membrane were fixed with pre-chilled (4°C) methanol and stained with crystal violet solution (0.1%, Sigma®). The images were acquired under inverted light microscopy with phase contrast (Nikon ECLIPSE Ti, Melville, New York, USA) and processed using Adobe® Photoshop® 7. For each well, the invaded cells in all visual fields observed at 200× magnification were manually counted. Prior to seeding the cells in the culture plate, cell viability was determined by exclusion of trypan blue dye. The experiments were conducted three times with comparable results.

## Supplementary Material

Supplementary Material
