# Supplementary material for: Aberrant over-expression of TRPM7 ion channels in pancreatic cancer: required for cancer cell invasion and implicated in tumor growth and metastasis
Source: Biol Open. 2015 Mar 13;4(4):507–14. doi: 10.1242/bio.20137088 (PMC4400593; doi:10.1242/bio.20137088)
Supplement: Supplementary Material [file supp_4_4_507__index.html]

Aberrant over-expression of TRPM7 ion channels in pancreatic cancer: required for cancer cell invasion and implicated in tumor growth and metastasis — Aberrant over-expression of TRPM7 ion channels in pancreatic cancer: required for cancer cell invasion and implicated in tumor growth and metastasis — Supplementary Material 

# Aberrant over-expression of TRPM7 ion channels in pancreatic cancer: required for cancer cell invasion and implicated in tumor growth and metastasis

## bio.20137088 Supplementary Material

**Files in this Data Supplement:**

- Supplementary Material - Nelson S. Yee et al. doi: 10.1242/bio.20137088
