# Supplementary material for: Aberrant over-expression of TRPM7 ion channels in pancreatic cancer: required for cancer cell invasion and implicated in tumor growth and metastasis
Source: Biol Open. 2015 Mar 13;4(4):507–14. doi: 10.1242/bio.20137088 (PMC4400593; doi:10.1242/bio.20137088)
Supplement: Supplementary Material [file supp_bio.20137088_bio.20137088-s1.pdf]

Biology Open (2015) 000, 1–8 doi:10.1242/bio.20137088

Supplementary Material
Nelson S. Yee et al. doi: 10.1242/bio.20137088

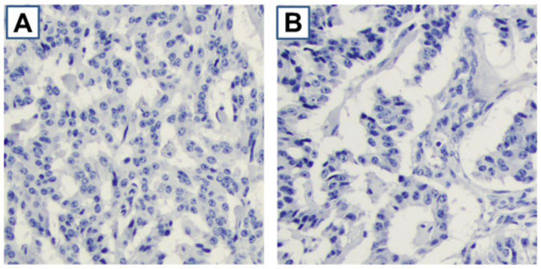

Fig. S1. Controls for anti-TRPM7 immunohistochemistry. Tissue sections were incubated with isotype matched non-specific primary antibodies (A) or secondary antibodies (B), and no TRPM7-specific immunoreactivity can be detected.

Biology Open
